# Theory-based approach to developing an implementation plan to support the adoption of a patient decision aid for Down syndrome prenatal screening

**DOI:** 10.1186/s13012-021-01103-5

**Published:** 2021-05-25

**Authors:** Titilayo Tatiana Agbadjé, Matthew Menear, Marie-Pierre Gagnon, France Légaré

**Affiliations:** 1 Canada Research Chair in Shared Decision Making and Knowledge Translation, Laval University, Quebec, QC Canada; 2 Centre de recherche en santé durable (VITAM), Québec, QC Canada; 3 Centre Intégré Universitaire de Santé et Services Sociaux de la Capitale-Nationale (CIUSSS-CN), Quebec, QC Canada; 4 Faculty of Nursing, Université Laval, Quebec, Canada; 5 Department of Family Medicine and Emergency Medicine, Faculty of Medicine, Université Laval, Quebec, Canada

**Keywords:** Decision aid, Shared decision making, Implementation plan, Knowledge-to-Action Framework, Theoretical Domains Framework, Participatory action research, IPDAS

## Abstract

**Background:**

Our team has developed a decision aid to help pregnant women and their partners make informed decisions about Down syndrome prenatal screening. However, the decision aid is not yet widely available in Quebec’s prenatal care pathways.

**Objective:**

We sought to identify knowledge translation strategies and develop an implementation plan to promote the use of the decision aid in prenatal care services in Quebec, Canada.

**Methods:**

Guided by the Knowledge-to-Action Framework and the Theoretical Domains Framework, we performed a synthesis of our research (11 publications) on prenatal screening in Quebec and on the decision aid. Two authors independently reviewed the 11 articles, extracted information, and mapped it onto the Knowledge-to-Action framework. Using participatory action research methods, we then recruited pregnant women, health professionals, managers of three prenatal care services, and researchers to (a) identify the different clinical pathways followed by pregnant women and (b) select knowledge translation strategies for a clinical implementation plan. Then, based on all the information gathered, the authors established a consensus on strategies to include in the plan.

**Results:**

Our knowledge synthesis showed that pregnant women and their partners are not sufficiently involved in the decision-making process about prenatal screening and that there are numerous barriers and facilitators of the use of the decision aid in clinical practice (e.g., low intention to use it among health providers). Using a participatory action approach, we met with five pregnant women, three managers, and six health professionals. They informed us about three of Quebec’s prenatal care pathways and helped us identify 20 knowledge translation strategies (e.g., nurse discusses decision aid with women before they meet the doctor) to include in a clinical implementation plan. The research team reached a consensus about the clinical plan and also about broader organizational strategies, such as training healthcare providers in the use of the decision aid, monitoring its impact (e.g., measure decisional conflict) and sustaining its use (e.g., engage key stakeholders in the implementation process).

**Conclusion:**

Next steps are to pilot our implementation plan while further identifying global strategies that target institutional, policy, and systemic supports for implementation.

**Supplementary Information:**

The online version contains supplementary material available at 10.1186/s13012-021-01103-5.

Contributions to the literature

- In Quebec (Canada), there are several options for Down syndrome (DS) prenatal screening. We had developed a decision aid (DA) to help pregnant women and their partners make informed decisions about DS prenatal screening.
- There is currently no guidance on how to implement a DA for DS prenatal screening in the different clinical pathways for prenatal care.
- We developed an implementation plan tailored to the different clinical pathways for prenatal care in Quebec.
- The next step will be to operationalize the implementation plan in the real-world clinical context to improve shared decision making about prenatal screening.

## Background

Screening for Down syndrome is becoming a part of routine prenatal care in a growing number of countries worldwide. In the province of Quebec, Canada, the provincial Trisomy 21 Prenatal Screening Program covers maternal serum screening tests and amniocentesis diagnostic tests for all pregnant women with public health insurance [1]. Other tests, such as nuchal translucency ultrasounds, are publicly covered in some circumstances [1]. Participation in this program is voluntary and pregnant women must decide whether or not to take these tests.

To help women and their partners make informed decisions consistent with the best available evidence and their preferences and values, health professionals can engage them in shared decision making (SDM) [2, 3]. One effective way to facilitate SDM is through the use of decision aids [4, 5]. Decision aids are tools (printed or digital) that can be used at point-of-care to provide information about a health condition, treatment options, and probabilities about possible benefits and harms [6, 7]. They help patients actively engage in decisions about their care and make choices that better match their values and preferences [5, 8]. In the context of genetic testing, pregnant women experience less decisional conflict [5, 9] and decisional regret [5] after using a decision aid. Yet, despite the proven benefits of decision aids, their use in routine clinical practice is limited [10, 11] and particularly rare in the context of prenatal care and screening for Down syndrome [12, 13].

Recently, our team developed and validated a decision aid for decisions about Down syndrome prenatal screening that respects the 16 criteria of the International Patient Decision Aid Standards (IPDAS) [14–16]. However, this decision aid has not yet been implemented in prenatal care settings. Given the many professional, patient-related, and contextual barriers to implementing SDM in routine clinical practice [17, 18], we felt it was necessary to develop a robust plan to promote adoption of the decision aid by the province’s prenatal services. Implementation planning is an important but understudied part of the process of implementing evidence-based practices [19]. The aim of this study was thus to create a theory-informed approach to developing an implementation plan to support the adoption of a patient decision aid for Down syndrome prenatal screening. Building on our previous work in this area and in consultation with stakeholders, we planned to identify the knowledge translation (KT) strategies needed to overcome barriers to implementing the decision aid in three of Quebec’s prenatal care pathways.

## Methods

### Conceptual framework

We used two conceptual frameworks from the field of implementation science: the Knowledge-to-Action (KTA) Framework [20, 21] and the Theoretical Domains Framework [22]. The KTA Framework describes the knowledge translation (KT) process as iterative, complex, and made up of two distinct but related components: knowledge creation and knowledge application (the action cycle). Knowledge creation is the production of knowledge that can be synthesized, refined, and converted into useful tools for end-users. The action cycle is based on planned action theories and consists of seven main phases: (1) identifying, reviewing, and selecting the knowledge to implement; (2) adapting or customizing the knowledge to the local context; (3) assessing the determinants of knowledge use; (4) selecting, tailoring, and implementing KT strategies; (5) monitoring KT strategies and knowledge uptake: (6) evaluating outcomes: and (7) determining strategies for sustained knowledge use [20, 23].

The Theoretical Domains Framework (TDF) provides a theoretical lens through which to understand factors influencing implementation and the determinants of behavior [24]. It represents a synthesis of 33 theories of behavior and behavioral change and features 84 theoretical constructs clustered into 14 domains. It has been used to explore barriers and facilitators to the adoption of evidence-based practices and to identify strategies to support implementation [24, 25]. Taken together, these theoretical frameworks provide guidance on the elements and strategies to include in an implementation plan.

### Study design and context

We performed a synthesis of our previous research on Down syndrome prenatal screening in Quebec. The synthesis included a review of articles related to several research projects, notably the CanGenTest and PEGASUS (PErsonalized Genomics for prenatal Aneuploidy Screening USing maternal blood) projects. The latter was an independent study to validate the performance and utility of this new genetic screening in Quebec’s public clinical laboratories and to enable pregnant women and their partners together with health professionals to make informed decisions about prenatal screening choices [26]. The funding for these projects was provided by a variety of federal and provincial agencies from 2008 until 2017.

We identified stakeholders involved in any aspect of the prenatal screening process: pregnant women, prenatal service health professionals and managers, and PEGASUS prenatal care researchers. Using the Participatory Action Research (PAR) approach, we asked the stakeholders to identify strategies and co-design an implementation plan specifically for the clinical context of prenatal care. PAR is a systematic inquiry, in collaboration of those affected by the issue being studied, for the purposes of education and taking action or effecting social change [27]. The stakeholders provided us with information not collected in our previous studies on the current organization of prenatal services in Quebec and on how our KT interventions could be tailored to the different clinical pathways. The stakeholders helped us identify the types of prenatal service in which most pregnant women in Quebec receive their care. Ethics approval for the PAR phase of the study was obtained from the research ethics boards of the Centre de Santé et de Services Sociaux de la Vieille-Capitale (#2013-2014-29) and the CHU de Quebec (#B14-02-1929).

We used the Standards for Reporting Implementation Studies (StaRI) checklist to report this study [28].

### Data collection

For the knowledge synthesis, two authors (TTA, MM) selected 11 articles published as part of our previous research. Articles were eligible if they related to decision-making or the implementation of SDM or decision aids in decisions about Down syndrome prenatal screening. The articles described results from: (a) a systematic review of the decisional needs of participants in Down syndrome prenatal testing [29], (b) a cross-sectional study examining the levels of SDM and decisional conflict during routine consultations about Down syndrome prenatal testing [13, 30, 31], (c) an environmental scan of decision aids for Down syndrome prenatal screening [32], (d) two mixed-methods studies examining pregnant women’s [33–35] and health professionals’ [36, 37] intentions to use a decision aid for Down syndrome prenatal screening, and (e) a theory-based qualitative study examining women’s perceptions of strategies to enhance the use of a decision aid for Down syndrome prenatal screening [38].

For the PAR phase of the study, we recruited managers knowledgeable about the organization of prenatal services in the province, end-users of our decision aid (pregnant women and health professionals), and researchers with expertise in Down syndrome prenatal screening. Prenatal service managers were recruited from three clinical sites in Quebec City: an academic family medicine clinic, a university hospital obstetrics/gynecology department, and a birthing center. One manager from each site was asked to provide details about the clinical pathways of pregnant women in their service. Meetings lasted 30 min and were conducted at the managers’ places of work.

With support from these managers, we then recruited pregnant women who had experience with each clinical pathway. Women were eligible if they were at least 18 years old, were no less than 16 weeks pregnant or had just given birth, had already made their decision about Down syndrome prenatal screening for their current pregnancy, and were able to read and speak French. We excluded women who had participated in previous phases of the PEGASUS project related to SDM, who presented a high-risk pregnancy (e.g., preeclampsia, gestational diabetes, multiple pregnancy), and whose delivery date corresponded with the dates of data collection. We drafted an initial list of 15 women recruited from each location and purposely selected five women to be partners in our study. The women were consulted individually at our research center during meetings lasting 30–45 min. This consultation focused on validating results of our previous study on strategies to enhance decision aid use by pregnant women [38], cross-checking their personal clinical pathway with the clinical pathways described by prenatal service managers, and eliciting their ideas for generating knowledge KT strategies.

We next approached health professionals who were staff members of the same three clinical sites. Two family physicians, a gynecologist, a midwife, a nurse, and a neonatologist agreed to participate. Meetings with the professionals focused on their perceptions of the appropriate strategies for promoting decision aid adoption in the different prenatal care pathways. Finally, we consulted four prenatal care researchers with longstanding involvement in the PEGASUS to explore their views on the KT strategies. The consultations with health professionals and researchers took place individually at their places of work and lasted approximately 30 min.

All consultations were conducted by one of the authors who was trained in public health and had experience in conducting qualitative research and who took detailed notes during meetings with stakeholders.

### Data analysis

Two authors (TTA, MM) reviewed the articles included in the knowledge synthesis. MM extracted barriers and facilitators from the 11 studies and categorized them in the TDF domains. Extraction and categorization were then verified by TTA. We chose to proceed in this way because both authors had prior knowledge of the studies and indeed had been involved in conducting several of them. Also, categorization was facilitated by the fact that five of the 11 studies used the TDF or a conceptual framework related to it. Following this, the two authors met to discuss any differences in interpretation, validated categorization, and established a consensus on the most relevant strategies for addressing barriers and facilitators, drawing on strategies found in our environmental scan and suggested in our stakeholder consultations.

For the PAR, TTA transcribed notes taken during interviews with stakeholders (pregnant women, health professionals, researchers, and clinical managers). Transcripts were categorized into themes according to the type of information sought (portrait of the clinical pathway, KT strategies generated, and views on KT strategies) and analyzed manually. TTA drafted a summary of the feedback from meetings with stakeholders. Then, authors discussed the summary and established a consensus on information that should be taken into account in the selection of KT strategies for the design of the implementation plan.

## Results

In the sections that follow, we use the elements of the KTA framework to describe what we learned about implementing a decision aid for prenatal screening from our knowledge synthesis and from our PAR study, and present the KT strategies identified. First, the “Knowledge Creation” section describes the knowledge we have acquired (through primary studies and reviews) and tools/products we have developed over the past 10 years. Second, we identify strategies for a plan to implement the decision aid in three clinical prenatal care contexts (steps 1–4 of the *Action Cycle*). Third, we identify broader-scale strategies for expanding/scaling up the implementation plan throughout the province of Quebec (steps 5–7 of the *Action Cycle*).

### Knowledge creation

#### Knowledge inquiry

Primary studies conducted by our team helped us generate valuable knowledge about the nature of decision-making about prenatal screening for Down syndrome and about the factors that influence decision aid adoption by pregnant women and health professionals. In particular, an initial cross-sectional study examining SDM for prenatal screening decisions in routine consultations revealed that women were rarely involved in these decisions and that any fears or concerns they may have had were rarely discussed [13, 31]. However, both women and health professionals were willing to engage in SDM, especially when they had a positive outlook on SDM and it was valued by significant others [31]. Subsequent mixed-methods and qualitative studies revealed that most pregnant women showed a similarly strong willingness to use decision aids to support their prenatal care decisions, though about a third (≈30%) were ambivalent [34]. Using the Theoretical Domains Framework as a guide, our team found that women’s intentions to use decision aids were influenced by a range of factors, including their attitudes and beliefs, their knowledge and skill levels, the nature of the decision aid itself, and the context in which they encountered it [34, 35]. Health professionals also generally showed strong intentions to use decision aids but this varied by type of professional, with midwives having the highest intentions and obstetrician/gynecologists having the lowest [37]. Professionals’ intentions to use decision aids were similarly influenced by many factors, including their attitudes and beliefs about the decision aidand their own professional identity, the compatibility and availability of the decision aid within their practice, and the use of decision aids by their peers [36, 37]. These studies thus reinforced the need for a multifaceted implementation approach that targeted pregnant women, their care providers, and the decision-making environments. The studies also shed light on potential facilitators and barriers to decision aid adoption that could be taken into consideration in the implementation plan (see below).

#### Knowledge synthesis

Our team had also conducted two knowledge syntheses to aggregate existing evidence on topics related to Down syndrome prenatal screening. From a first systematic review on the decisional needs of pregnant women, their partners, and health professionals [29], we learned that in some cases participants found prenatal screening decisions to be difficult because of anxiety or fear, lack of information available about risks and benefits, and the possibility that they would have to make decisions that contradicted their personal values. In other cases, prenatal screening had not been presented to the women as a topic to discuss but rather as a part of routine care without any decision needed. Decision-making was made easier when women had better access to information, personal or professional support, and were clear about their personal values around pregnancy and childbirth. This review also highlighted the important role of women’s partners, who often participate in decisions and can be important sources of support or pressure in decision-making. In a second knowledge synthesis, an environmental scan was performed to identify publically available decision aids that focus on prenatal screening or diagnosis of Down syndrome [32]. We identified 20 decision aids, including five for prenatal screening only, three for prenatal diagnosis, and 12 that covered both screening and diagnosis. However, none of them met all the IPDAS criteria to qualify as high-quality, effective decision aids, indicating a clear need for new knowledge tools on these topics.

#### Knowledge tools/products

Following the environmental scan, we developed a decision aid to help pregnant women and their partners make screening decisions [32]. This decision aid was adapted from another identified in the scan that had scored relatively well on the IPDAS criteria (10 out of 16 criteria) and that seemed relevant to the Quebec context. The new decision aid met all minimal IPDAS criteria and was available in French and English. It was also the foundation for two other products, namely a video illustrating the use of an SDM approach in the context of Down syndrome prenatal screening (in which the decision aid appears) and an online training program for health professionals to improve their SDM skills in this context.

### Strategies for a clinical implementation plan

#### Action cycle step 1: identify the problem and the knowledge to be implemented

Making decisions about Down syndrome screening can be challenging given its important consequences for the child and family. Yet pregnant women and their partners may not be involved in the decision-making process, may lack information to help inform their decisions, or in some cases may not even be informed there is a decision to make. In 2017, the Quebec government published an evaluation that confirmed the findings of our research projects [39]. Among women participating in the provincial Trisomy 21 Prenatal Screening Program, only 61% of women received written materials about the tests, 34% received no information about the diagnostic amniocentesis test and its risks, and 50% received no information on the options of pursuing or terminating their pregnancy in the event of prenatal diagnosis of Down syndrome [39]. When women lack information about the options, not only are they deprived of their right to informed consent [39], but there is a high risk that they will experience decisional conflict [40] and decisional regret [30]. We thus identified the main problem as an absence of SDM and decision support for people facing Down syndrome screening decisions, prompting us to develop the new decision aid for use in prenatal services. The barriers to decision aid adoption in routine care identified in our research also highlighted the need for a multi-faceted plan for supporting implementation that would take environmental context, policies, training, and health system organization into account.

#### Action cycle step 2: adapt knowledge to local context

**Fig. 1:**
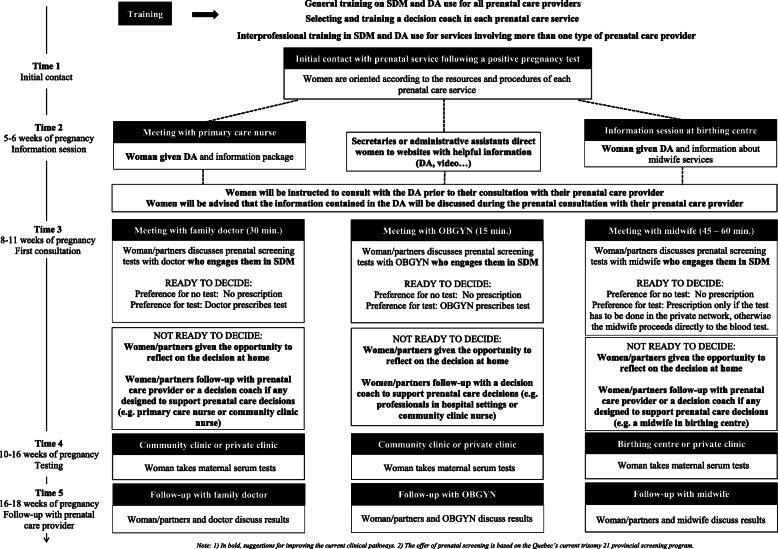
General training on SDM and decision aid use for all prenatal care providers. Selecting and training a decision coach in each prenatal care service. Shared decision making in the context of Down syndrome

Our consultations with managers, health professionals, researchers, and pregnant women during the PAR process shed important light on the contexts of prenatal care in Quebec and the different clinical pathways followed by pregnant women (Table 1). The stakeholders from the various prenatal care services helped us understand how to adapt knowledge to these different clinical contexts. Implementation of the decision aid would be more straightforward in birthing centers because midwives expressed the highest intention to use it, their consultations were generally longer, and there was more flexibility in the care pathway to allow women and their partners to reflect on the decision. In contrast, implementing the decision aid among obstetrician/gynecologists in a hospital setting would take more finely tuned strategies as they had less favorable attitudes towards decision aids, their consultations were shorter, and they were less surrounded by other professionals who could assist with decision support (Fig. 1).

**Table 1:** Clinical pathways for pregnant women in Quebec

Activity	Family medicine clinic	Hospital obstetrics/gynecology department	Birthing centre
Initial contact with service	• Following a positive pregnancy test, women make contact with the clinic for a prenatal consultation. • Women are scheduled to meet with a nurse for 1 h after 5–6 weeks of pregnancy. • At that meeting, women are provided with an information kit that includes a brochure created by the government on Down syndrome prenatal screening.	• Following a positive pregnancy test, women make contact with the obstetrics/gynecology service for a prenatal consultation.	• Following a positive pregnancy test, women make contact with the birthing center for a prenatal consultation. • Women are invited to attend an evening information session to learn about midwifery care. • Only women with low-risk pregnancies are eligible for this service. • Women are contacted after 2–3 weeks to let them know if they have been assigned a midwife or are on a waiting list.
Initial contact with main prenatal care provider	• Women meet with their physician after 8–9 weeks of pregnancy.	• Women meet with a gynecologist after 9–11 weeks of pregnancy.	• Women meet with the midwife after 10–11 weeks of pregnancy.
Prenatal screening process	• At the initial meeting with the physician, the topic of Down syndrome prenatal screening is discussed. • If the decision to take the maternal serum tests is made right away, the physician writes a prescription for the test. • For women who need more time to think about the decision, the physician writes an open prescription for the test. • Women make an appointment to take the first serum test at a local community health center or a private clinic in weeks 10–13 of their pregnancy. • After the first serum test, women make an appointment for the second serum test in weeks 14–16 of their pregnancy.	• At the initial meeting with the gynecologist, the topic of Down syndrome prenatal screening is discussed. • If the decision to take the maternal serum tests is made right away, a form is completed to prescribe both tests. • Women make appointments to take the first serum tests at a local community health center or a private clinic during weeks 10-13 of their pregnancy. • During that appointment, women will meet a nurse for one hour and discuss topics such as diet and physical activity during pregnancy. • After the first serum test, women make an appointment for the second serum test in weeks 14–16 of their pregnancy.	• At the initial meeting with the midwife, the topic of Down syndrome prenatal screening is discussed. • The midwife presents the government’s brochure on Down syndrome prenatal screening and explains women’s options. • If the decision to take the maternal serum tests is made right away, the midwife administers the test at the birthing center during the same meeting. • If women need more time to think about the decision, they can return home, call the birthing center for advice, and make another appointment with the midwife within the next few weeks. • Women are scheduled to meet the midwife again after 14–16 weeks of pregnancy for another consultation and the second serum test.
Prenatal screening results announcing process	• Usually, the result is available 3–7 days after the second test. • If the result is low-risk, the doctor waits until the next visit and gives them the result in the usual sequence. • If the risk is high, the doctor calls the woman to inform her of the result and its meaning. The doctor recommends a genetic consultation to get a complete risk history and recommends further testing. • The next meeting with the doctor is between 16 and 20 weeks of pregnancy.	• Usually, the result is available 3–5 days after the second test. • Some OBGYNs call regardless of the result and others call only if the result is high-risk (there is a variation in practices). • Women are scheduled for another visit as soon as possible if result is abnormal. If normal, usually around 20 weeks to review the results of the tests and follow-up.	• Usually, the result is available 3-4 days after the second test. • The midwife calls the woman as soon as she receives her test result, whether the woman is at risk or not. • When the risk is high, the midwife transfers the woman’s file to the geneticist.
	Private testing services notify the patient if normal but if abnormal, refer them to their prenatal care provider.

#### Action cycle step 3: assess barriers/facilitators to knowledge use

Barriers and facilitators to the adoption of decision aids in prenatal care were initially identified through the knowledge synthesis and through the PAR consultations. We categorized them in our implementation plan according to their TDF domains (Table 2). We selected KT strategies that might be taken to address them, identified in the next step, to these domains. For instance, for women dealing with anxiety, in the TDF domain “emotion,” the strategy might be to have a decision coach accompany women when they use the decision aid and be reassured that their questions will be answered by a health professional.

#### Action cycle step 4: select, tailor, and implement interventions

The selection of further KT strategies was informed by both the knowledge synthesis and the PAR consultations. Among the studies in our knowledge synthesis was a qualitative study that explored pregnant women’s views on strategies for promoting the use of a decision aid for Down syndrome prenatal screening [38]. This study revealed several strategies that women perceived as relevant and acceptable for supporting decision aid adoption, including (1) using credible sources (e.g., receiving it directly from their prenatal care providers or retrieving it from hospital or government websites), (2) goal setting around the use of the decision aid (e.g., having professionals clearly explain its purpose), (3) ensuring decisional and social support (e.g., supporting women through the decision-making process, answering their questions, including women’s partners in the decision), (4) demonstrating the use of the decision aid (e.g., showing women the video illustrating its use), (5) modifying the environment in which it is to be used (e.g., ensuring its availability for clinicians and placing posters and messages on screens in the waiting rooms of clinics encouraging its use).

We selected an initial set of KT strategies, including those from step 3. We then invited our stakeholders (pregnant women partners, healthcare professionals, and PEGASUS project members) for a second round of consultation during which they gave their feedback on the strategies and proposed implementation plan. Some of the strategies applied to all three prenatal contexts while others were tailored to each of the different prenatal care contexts. The final set of KT strategies is presented in Table 2 and the final plan is presented in Fig. 1. Based on the Effective Practice and Organisation of Care (EPOC) taxonomy, we used the strategies to propose an implementation plan for shared decision making in the context of Down syndrome prenatal screening (Table 3) [41].

**Table 2:** Barriers and facilitators to the use of decision aids for Down syndrome prenatal screening and knowledge translation strategies as suggested by stakeholders

TDF domain	Facilitators	Barriers	Strategies
Social influences	- Colleagues’ approval of the use of DAs - Women’s partners’ approval of the use of the DA - Women’s providers’ approval of the use of the DA	- Colleagues’ disapproval of the use of DAs - Women’s partners’ disapproval of the use of the DA - Women’s friends’ disapproval of the use of the DA	- Establish a multi-stakeholder steering committee to guide the implementation project that includes different health professionals involved in prenatal care, health managers, policymakers, researchers, and pregnant women and their partners - Develop a communication plan that promotes shared decision making and the use of the DA among prenatal care providers - **Develop a tailored communication strategy to promote the use of the DA for health professionals working in hospital settings**
Social/professional role and identity	- Providers feel that it is their duty to provide the DA to women - Providers feel that using the DA is consistent with best practices - Not feeling forced to use the DA in all circumstances	None	
Environmental context and resources	- Having the DA available in the provider’s office - Having the DA freely available on a website - Having Down syndrome risk factors in the family	- Not having the DA available in the provider’s office - The DA is available only in printed form - Not having time to present the DA during a visit	- Make the DA freely available on credible websites - Supply paper copies of the DA to prenatal care providers - Ensure that brochures and posters on prenatal care decision making are available for patients in clinic waiting rooms
Memory, attention and decision processes	- The DA is presented and explained to women before their consultation with their main provider - DA viewed as promoting decision-making	- DA presents too much information - DA content is too complex for patients - DA content is incomplete - The provider presents the DA in an unconvincing or uninteresting way	- Review DA content and ensure that its evidence is up-to-date - Review the language used in the DA and consider issues of health literacy, numeracy and risk communication - Improve the graphic design of the DA and ensure an acceptable balance between length and completeness of information
Beliefs about capabilities	- Providers feel comfortable using the DA - The DA increases decision-making competencies	None	- Encourage health professionals to complete the online training modules on shared decision making and the use of DAs in prenatal care - Make the video on the use of the DA easily accessible to patients online - **Provide in-person training in inter-professional approaches to shared decision making to health professionals in hospital settings** - **Provide training to professionals in hospital settings to perform the role of decision coaches who will work with women, their partners, and OBGYNs to support prenatal care decision-making**
Knowledge	- DA viewed as a relevant source of information	- Lack of knowledge about DAs and how to use them	
Skills	- Training in the use of DAs	- Lack of health literacy in some women	
Intentions	- Intentions of women to use the DA are high - Intentions of some providers (e.g., midwives) to use the DA are high	- Intentions of some providers (e.g. OBGYN) to use the DA are lower	
Motivation and goals	- Use of the DA consistent with women’s need to be informed	None	- Ensure that women receive the DA from a health professional early in the course of their prenatal care (e.g., during information sessions) before they meet with their main prenatal care provider; or - Ensure that couples receive the DA (from administrative assistant, secretary or nurse) prior to their first prenatal consultation with their health professional; and specify that this will be discussed at the meeting with the health professional. - Encourage prenatal care providers to use the DA during consultations to support prenatal care decisions - Encourage prenatal care providers to suggest to women that they reflect on their decisions at home with their partners - **Identify a OBGYN who can champion shared decision making and the use of the DA in hospital settings** - Make sure the women are accompanied when they consult the DA, or know that their questions will be answered by a health professional - Name a decision coach to discuss and present the DA before the consultation
Beliefs about consequences	- Belief that the DA helps the women to think about the decision - Belief that the DA enables women to express their preferences - Belief that the DA helps the couple reflect on decisions together at home - Belief that the DA helps women make informed decisions - Belief that the DA reduces decisional regret - Belief that the DA’s visual content is helpful for patients	- Belief that the DA could create confusion during the decision-making process	
Emotion	- Feelings of satisfaction when using the DA	- The DA induces stress by increasing knowledge or risks and benefits of the tests - The DA raises fears about the results of the tests	
Behavioral regulation	None	None	N/A
Reinforcement	None	None	N/A
Optimism	None	None	N/A

Note: Strategies in bold indicate strategies tailored to the individual prenatal care settings. *DA* Decision aid

While our initial focus was on strategies appropriate for an implementation plan at the organizational level of clinical prenatal care pathways, the following strategies identified in our knowledge synthesis are also appropriate for a long-term province-wide implementation plan.

### Strategies for a long-term province-wide implementation plan

#### Action cycle step 5: monitor knowledge use

Monitoring knowledge use in real time will be critical in order to determine whether our KT strategies are sufficient to bring about desired changes, i.e., whether women and their prenatal care providers are adopting the decision aid and engaging in SDM. As a research team, we identified several low-cost monitoring strategies to integrate into the clinical implementation plan, including (1) monitoring the distribution of paper copies of the decision aid and the number of online downloads of the decision aid, (2) monitoring the distribution of posters to the different clinical settings, and (3) tracking the number of professionals completing the online and in-person training on SDM and decision aids. In select pilot implementation sites, clinician-patient encounters could be video recorded to examine the adoption and fidelity of decicion aid use. Using multiple data sources over longer time periods is also necessary to explore the sustainability of their use in different clinical care contexts (Table 4).

**Table 3:** An implementation plan for shared decision making in the context of Down syndrome prenatal screening

Categories (EPOC categories)	Strategies to be implemented
**Delivery arrangements**	
***Coordination of care and management of care processes***	
Care pathways	Integrate into existing clinical pathways distribution of the DA, reading it, and discussing it with the health professional.
Shared decision-making	Prenatal care providers involve pregnant women in shared decision-making in their clinical practice and encourage their active participation in decision-making.
Continuity of care	In obstetrics-gynecology departments, assign follow-up prenatal screening for women who need more support to a designated decision coach.
**Implementation strategies**	
***Interventions targeting healthcare organizations***	
Organizational culture	- As this is not yet part of the organizational culture of prenatal care services, establish a multi-stakeholder steering committee to guide implementation that includes all types of health professionals involved in prenatal care, health managers, policymakers, researchers, and pregnant women and their partners. - Develop a communication plan that promotes shared decision making and the use of the DA among prenatal care providers. - Identify an OBGYN who can champion shared decision making and the use of the DA in hospital settings.
***Interventions targeting healthcare workers***	
Educational materials	Distribute educational material to health professionals for the use with the woman and her partner during consultation, including paper-based DA.
Educational meetings	- Provide prenatal care providers with access to existing online training modules on shared decision making and the use of DAs in prenatal care. This training aims to improve their knowledge and skills on shared decision making, Down syndrome prenatal screening, decision aids, and communication between healthcare professionals and patients. - Identify appropriate health professionals in hospital settings to train as decision coaches who will work with women, their partners, and OBGYNs to support prenatal care decision-making.
Inter-professional education	Provide health professionals in hospital settings with in-person training on inter-professional approaches to shared decision making.
Tailored interventions	Create a communication strategy tailored to promoting the use of the DA among health professionals working in hospital settings.
***Interventions targeting patients***	
Distribution and use of decision aid	- Distribute DA on DS prenatal screening to all pregnant women. - Make available free web-based version of the DA on credible websites. - Make available video on the use of the DA to patients online for women with low literacy skills and for people who prefer animated to a written material. - Invite each pregnant woman who receives the DA to consult it, to write down her questions and discuss them with her health care professional during the consultation.
Reminders	Develop and display posters in waiting room and consultation rooms. For services with a television screen in their waiting room, display messages on screen.
Routine patient-reported outcome measures	Following the use of the DA, collect patient-reported outcome measures such as such as knowledge, decisional conflict, decision regret, involvement of partner, and satisfaction among women who used it to make prenatal screening decisions.

*EPOC* Effective Practice and Organisation of Care; *DA* Decision aid

**Table 4:** Strategies for province-wide application of the implementation plan (KT action steps 5–7)

KTA action cycle step	Strategies
*Monitor knowledge use*	• Monitor distribution of paper copies of the DA and the number of online downloads of the DA • Monitor distribution of posters and brochures to clinical settings province-wide • Track the number of professionals completing the online and in-person training on SDM and DAs • Video recordings of patient-physician encounters to examine the adoption and fidelity of DA use
*Evaluate outcomes*	• Surveys to assess participants’ behavioral intention to use DAs or engage in SDM • Regularly measure SDM outcomes such as knowledge, decisional conflict, decision regret, knowledge, involvement of partner, and satisfaction among women making prenatal screening decisions
*Sustain knowledge use*	• Engage policymakers to integrate DA into provincial prenatal screening program • Engage key stakeholders in implementation plan • Make DA freely available in multiple formats and through multiple platforms • Provide low-cost online training programs throughout the provincial healthcare education system • Train for decision coaches in each clinical setting • Embed measures/indicators in clinical and information systems

DA - Decision aid

#### Action cycle step 6: evaluate outcomes

In Quebec, more than 80,000 births were registered for the year 2018 [42]. Reliable statistics on the number of women who undergo Down syndrome prenatal screening each year are not available, likely because of the mix of public and private testing options. However, integration of the prenatal screening decision aid into clinical practice is not intended to change screening rates but rather to increase women’s involvement in decision-making and support them in achieving informed, shared decisions that reflect their values and preferences. Our previous studies in this area shed light on a variety of relevant outcomes for evaluating the success of our KT strategies: first, assessing behavioral intentions regarding adoption among women and healthcare providers [31, 34, 37]; second, measuring the influence of decision aid adoption on women’s involvement in prenatal screening decisions and in their experiences of decisional conflict [43]; third, measuring women’s knowledge of their options, their satisfaction with their decision and the extent to which it aligns with their values and preferences [44–46]; and finally, assessing the involvement of women’s partners in prenatal care decisions [46]. In a pilot phase, we will measure all these outcomes and demonstrate the impact of the implementation plan on adoption of the prenatal screening decision aid at the organizational level, see Table 4.

#### Action cycle step 7: sustain knowledge use

The notion of sustaining evidence-based practices over time has received growing attention in recent years [47–49], and we conceived our implementation plan with sustainability in mind. It includes six sustainability strategies. First, engagement of key stakeholders in the implementation process so that they share responsibility for increasing the involvement of women and their partners in their prenatal care. Second, the decision aid will be permanently and freely available through multiple platforms and its content and design will be repeatedly reviewed and updated to ensure it continues to be relevant. Third, our project steering committee plans to work with policymakers to promote the decision aid as an integral part of the province’s prenatal screening program. Fourth, our training strategies use low-cost existing online training modules that have been tested in a variety of clinical contexts. Fifth, our team continues to work with health organizations to provide training on interprofessional approaches to SDM, including on the use of this decision aid. Sixth, we plan to work with partners in hospital settings to train practising professionals as prenatal decision coaches, including in the use of this decision aid, using train-the-trainer models to mitigate potential staff turnover. Finally, to ensure ongoing and sustainable outcomes evaluation, we will work with stakeholders to embed further measures and indicators within the clinical and information systems used by providers (Table 4).

## Discussion

The use of decision aids to support shared decisions about Down syndrome prenatal screening is not a widespread practice in Quebec. Our team aimed to change this. Using two conceptual frameworks drawn from implementation science, we developed tailored KT strategies and a plan for implementing the new decision aid in three common prenatal care settings. To our knowledge, our paper is among the first to describe in detail the process used to develop such an implementation plan, a critical step in the process of implementing SDM.

Our plan addresses clinic-level organizational change at every stage, i.e., from training health professionals and raising patient awareness to following up with women post-decision. Though there is now extensive literature on SDM and decision aids, there is still uncertainty about how to implement them as sustainable practices in routine care [50, 51]. Passive dissemination strategies fail to address challenges stemming from professional attitudes and identities, lack of training, and organizational inertia [52], resulting in low levels of implementation and sustainability [52]. Several authors now argue that multi-faceted interventions targeting multiple levels (e.g., service users, providers, teams, organizations, systems) and addressing a range of facilitators and barriers are likely to be more successful [52–55]. For example, a robust attempt to implement SDM in routine care took place in the UK with the MAGIC program, a 3-year-quality improvement initiative (2010–2013) seeking to embed SDM within multiple clinical areas across a range of primary and secondary care settings [56, 57]. Investigators spent over 15 months designing and testing strategies and interventions to support the dissemination, adoption, and sustainability of SDM using the following strategies: (a) training and performance feedback activities, (b) support through the use of decision aids, (c) marketing campaigns, (d) facilitation and peer support, (e) increasing patient awareness and involvement, and (f) institutional supports for implementation [56, 57]. The result was improvements in care providers’ SDM-related knowledge and skills and increased use of decision support materials. The program also led some clinical teams to integrate SDM into their clinical routines, although results were not consistent in all clinical settings and some providers remained ambivalent about the need to share decisions with patients [56, 57]. The MAGIC study underscores the complexity of the implementation process, the diversity of potential barriers, and the need for bundles of KT interventions working together holistically to achieve broad and sustainable practice changes [54]. It also underscores how iterative the process of planning and implementation needs to be in real-world clinical settings.

Our process of establishing a clear and comprehensive user-designed implementation plan before moving to implementation itself may improve the effectiveness and efficiency of the change process. However, there is little guidance available on what the components of implementation plans should be, the steps involved in their development, and how to select the most appropriate KT strategies [19, 58]. Meyers and colleagues’ implementation plan involves needs assessments and readiness assessments, adapting the innovation to context, obtaining buy-in from key stakeholders, building organizational capacity, providing effective training, and creating implementation teams [59]. For their part, Damschroder et al. [19] suggest assessing stakeholders’ needs and perspectives, tailoring strategies, delivering information and education, establishing communication channels, tracking progress, and preparation with simulations or trial sessions. While studies of SDM implementation are rarely theory-informed [52], Damschroder et al. also propose basing their plan on behavioral or organizational theory. In the present study, stakeholder assessments using the PAR approach as well as the KTA and TDF frameworks helped us identify key strategies for our plan. The details of the plan were informed by our team’s work since 2008 in exploring attitudes around Down syndrome prenatal care screening, intentions to use decision aids, and facilitators and barriers to their use. We also developed contacts with key stakeholders who will facilitate its future adoption in prenatal care contexts in Quebec.

Another distinguishing feature of our process was our efforts to understand the clinical contexts and pathways associated with DS prenatal screening and care. The importance of understanding context has been cited frequently in the literature on implementation [60–62] and other work on decision aid implementation emphasizes taking clinicians’ workflows into account [57]. Using the PAR approach, we learned that hospital-based settings were likely to be the most challenging for decision aid implementation. We thus included five KT strategies tailored to the hospital context that target professional identities, attitudes, competencies, and capabilities. We expect that buy-in from senior hospital leaders and champions will be critical to the success of our implementation plan and will improve SDM with women cared for by gynecologists.

The present study has several strengths and limitations. Among the strengths was adoption of a theory-informed and participatory approach to developing our implementation plan, which included in-depth knowledge of the three main prenatal care pathways in the province. One limitation may be that our knowledge synthesis did not include studies beyond those conducted by our research team. This likely limited our access to information and lessons learned from other investigations into the implementation of decision aids in the context of prenatal care or screening practices. However, our research team has been an international leader in this area and we were able to draw on 11 articles representing over a decade’s work on this topic, which provided our team with useful and context-specific knowledge to inform our plan. With respect to our PAR study, we had a limited number of participants and did not gather the perspectives of community-based nurses, who are also involved in supporting prenatal screening decisions in primary care and other community settings. In preparation for a province-wide implementation plan, a broader coalition of stakeholders will be sought for the pilot phase of the study, which will involve operationalizing the implementation plan and its interventions, determining which sites will participate, and timelines for implementation activities.

## Conclusion

In this study, we propose KT strategies and a clinical implementation plan for promoting use of a decision aid by pregnant women for the decision to undergo or not DS prenatal screening. Tailored for use in three clinical prenatal care settings, this theory-informed and user-designed implementation plan is intended to help institutionalize the practice of shared decision making for the difficult decision of DS prenatal screening. It ultimately aims to improve the way clinicians engage pregnant women and their partners in the decision-making process, and ensure that decisions better reflect their values and preferences. Our implementation plan could be integrated into the Quebec government’s DS Prenatal Screening Program and, with adaptations, to that of other governments. Next steps are to pilot our implementation plan while further developing global strategies that target institutional, policy, and systemic supports for implementation.

## Supplementary Information

**Additional file 1.** Standards for Reporting Implementation Studies (StaRI) checklist.

## Data Availability

The datasets used and/or analyzed during the current study are available from the corresponding author on reasonable request.

## Abbreviations

DS: Down syndrome
DA: Decision aid
SDM: Shared decision making
KTA framework: Knowledge to action framework
TDF: Theoretical domains framework
BCT: Behaviour change techniques
PEGASUS: Personalized Genomics for Prenatal Aneuploidy Screening Using Maternal Blood
KT: Knowledge translation
